# Tuberculosis contact investigation following the stone-in-the-pond principle in the Netherlands – Did adjusted guidelines improve efficiency?

**DOI:** 10.2807/1560-7917.ES.2021.26.45.2001828

**Published:** 2021-11-11

**Authors:** Sarah van de Berg, Connie Erkens, Christiaan Mulder

**Affiliations:** 1 KNCV Tuberculosis Foundation, The Hague, the Netherlands; 2 Amsterdam Institute for Global Health and Development, Amsterdam University Medical Center, Amsterdam, the Netherlands

**Keywords:** tuberculosis, contact investigation, stone-in-the-pond, surveillance data, yield, coverage, efficiency, evidence-based guidelines

## Abstract

**Background:**

In low tuberculosis (TB) incidence countries, contact investigation (CI) requires not missing contacts with TB infection or disease without unnecessarily evaluating non-infected contacts.

**Aim:**

We assessed whether updated guidelines for the stone-in-the-pond principle and their promotion improved CI practices.

**Methods:**

This retrospective study used surveillance data to compare CI outcomes before (2011–2013) and after (2014–2016) the guideline update and promotion. Using negative binomial regression and logistic regression models, we compared the number of contacts invited for CI per index patient, the number of CI scaled-up according to the stone-in-the-pond principle, the TB and latent TB infection (LTBI) testing coverage, and yield.

**Results:**

Pre and post update, 1,703 and 1,489 index patients were reported, 27,187 and 21,056 contacts were eligible for CI, 86% and 89% were tested for TB, and 0.70% and 0.73% were identified with active TB, respectively. Post update, the number of casual contacts invited per index patient decreased statistically significantly (RR = 0.88; 95% CI: 0.79–0.98), TB testing coverage increased (OR = 1.4; 95% CI: 1.2–1.7), and TB yield increased (OR = 2.0; 95% CI: 1.0–3.9). The total LTBI yield increased from 8.8% to 9.8%, with statistically significant increases for casual (OR = 1.2; 95% CI: 1.0–1.5) and community contacts (OR = 2.0; 95% CI: 1.6–3.2). The proportion of CIs appropriately scaled-up to community contacts increased statistically significantly (RR = 1.8; 95% CI: 1.3–2.6).

**Conclusion:**

This study shows that promoting evidence-based CI guidelines strengthen the efficiency of CIs without jeopardising effectiveness. These findings support CI is an effective TB elimination intervention.

## Introduction

The Netherlands is a low tuberculosis (TB) incidence country with 4.7 new TB patients per 100,000 population; in 2018, 806 patients were notified [1]. Since the 1980s, contact investigation (CI) has been one of the pillars of TB control and is considered essential for the prevention of outbreaks and transmission [2,3]. In high burden and low resource settings, CI focusses primarily on TB screening of people living with HIV (PLHIV), children younger than 5 years old [4], and household and close contacts of index patients with sputum smear-positive pulmonary TB or drug-resistant TB (DR-TB). In low burden and high resource settings such as the Netherlands, CI takes on a broader focus, which includes identifying other exposed contacts, contacts of sputum smear-negative patients and the transmission source of TB patients who are likely to have been recently infected (source or reverse CI) [3,5]. The Dutch guidelines recommend conducting a CI of potentially infectious TB patients [6], i.e., patients with culture-confirmed pulmonary TB and with extrapulmonary TB where transmission may have occurred. Source investigation should be considered for all recently infected TB patients if the source patient is unknown or not diagnosed and likely traceable in the Netherlands.

CI should use a sequence of priority decisions to identify all contacts with active TB or latent TB infection (LTBI) without screening non-exposed or low risk contacts as such efforts would be an inappropriate use of public resources [2]. In the Netherlands, CI is conducted according to the stone-in-the-pond principle: contacts are prioritised for testing in concentric circles around the index patient, depending on the level of exposure and vulnerability of the contact, until the prevalence of infection approximates that of the local community [7].

Since 2006, CI results have been recorded in the national TB surveillance registry. This registry allows for the monitoring and evaluation of national policy and the performance of the Public Health Services (PHSs) responsible for conducting the CI. Evaluation of the data between 2006 and 2010 showed that CI had an active TB yield of 0.4% and a LTBI yield of 5% [8]. During this period, however, the testing coverage for LTBI was low (73%) as BCG-vaccinated contacts and contacts from high burden countries were not eligible for LTBI testing until 2010, when interferon gamma release assays (IGRAs) were recommended for use in these populations. Qualitative research showed that the national guidelines were not followed completely, and public health nurses did not fully adhere to the stone-in-the pond principle [9]. Based on these findings, it was deemed likely that the TB and LTBI yields could be increased by improving the targeting of individuals eligible for CI through the stone-in-the-pond principle and by providing LTBI testing for BCG-vaccinated contacts and contacts from high burden countries. CI guidelines [6] were updated accordingly in 2013 [8]. Dissemination and implementation of the guideline changes were supported through the development of operational guidance and tools as well as nation-wide 2-day multidisciplinary on-site trainings of all healthcare staff of the PHSs involved in CI of TB. The training is mandatory for TB nurses and physicians working at the PHSs and is offered on an annual basis to all new professionals.

The objective of this study was to determine whether the guideline adaptation in 2013 resulted in more efficient but equally or more effective CI practices by determining whether there was a decrease in the number of contacts being invited for CI per index patient, an increase in the number of CI scaled-up according to the stone-in-the-pond principle, and an increase in TB and LTBI testing coverage while the relative yield of active TB and LTBI remained similar or increased.

## Methods

This retrospective cohort study used records of TB patients registered in the Netherlands Tuberculosis Register (NTR) between 1 January 2011 and 31 December 2016. Records were included if a CI was initiated. Patients with incomplete or inconclusive CI data were excluded. If a CI had more than 200 invited contacts, it was considered an outlier and therefore excluded. The efficiency and effectiveness were compared between CI of patients registered between 1 January 2011 and 31 December 2013 (‘before the guideline adaptation’) and CI of patients registered between 1 January 2014 and 31 December 2016 (‘after the guideline adaptation’). We compared the number of contacts invited for CI per index patient, the number of CI scaled-up according to the stone-in-the-pond principle, the TB and LTBI testing coverage, as well as the relative yield of active TB and LTBI.

According to national guidelines, the first priority group of contacts include those considered most exposed to the index patient (household contacts and other close contacts) as well as vulnerable contacts (children younger than 5 years old and immunocompromised persons). Priority contacts are determined by PHS staff based on information collected from the index patient during a personal interview [6] (Supplement S1). Proof of transmission is defined as a contact identified with active TB, a child younger than 5 years old with LTBI, or prevalence of LTBI among evaluated contacts at least twice as high than the expected background LTBI prevalence based on country of origin and age (Supplementary Table S2). When the number of identified close contacts is too small to properly examine transmission, it is common practice to include a subgroup of casual contacts who are considered second most exposed to the index patient [6]. The screening algorithm for identified contacts is presented in Supplementary Table S3.

The coverage of contacts tested for TB was defined as the number of contacts tested for TB divided by the number of contacts invited for CI. The coverage of contacts tested for LTBI was defined as the number of contacts tested for LTBI (tuberculin skin test (TST) and/or an IGRA) divided by the number of contacts invited for CI. The yield of TB and LTBI was defined as the number of contacts identified with TB and LTBI, respectively, divided by the number of contacts tested for TB and LTBI, respectively. LTBI was defined as being TST and/or IGRA positive according to the national guidelines. Because the NTR data are aggregated per index patient, individual contact-based data were not available.

### Statistical analysis

The number of contacts invited per index patient before and after the guideline adaptation were compared using negative binomial regression. The number of CI where the stone-in-the-pond principle was appropriately applied was compared using logistic regression. To correct for the effect of the number of close contacts on the appropriate scale-up to casual contacts, the number of close contacts investigated per CI was included in this model as covariate.

TB and LTBI coverage and yield in the two periods were compared using generalised estimating equations (GEE) logistic regression model. We treated each index patient as a cluster as the NTR reports the number of contacts tested for TB/LTBI and identified with TB/LTBI aggregated per index patient. Models on number of contacts invited per index patient, coverage and yield were a priori stratified by priority of contact.

The following characteristics of the index patients were included as covariates and assessed for all models: sex; age (0–14; 15–29; 30–44; 45–59; 60–74; 75 + ); infectiousness (smear-positive pulmonary TB, smear-negative, culture-positive pulmonary TB, smear-negative, culture-negative pulmonary TB and extrapulmonary TB); ethnicity (Dutch or non-Dutch); belonging to a marginalised group (individuals who are homeless, addicted to drugs or addicted to alcohol); and reason for examination – active (i.e., identified through screening) or passive (i.e., identified in clinical care through presentation of symptoms).

Covariates with a univariate p value ≤ 0.2 were included in the multivariable models. Subsequently, the most parsimonious model was selected by backward elimination guided by the change and coefficients and log likelihood, if applicable, of successive models. A p value ≤ 0.05 was regarded as statistically significant. All statistical analysis were performed in SPSS version 25.0 (SPSS, Chicago, IL, United States).

### Ethical statement

The NTR Registration Commission approved the use of the NTR data. Ethical approval was not required as the data were anonymised and aggregated retrospective surveillance data.

## Results

Between 2011 and 2016, 5,368 patients were registered in the NTR. After cleaning the data and applying the selection criteria, 3,192 index patients were included in the analyses – 1,703 before and 1,489 after the guideline adaptation (Supplementary Figure S4). Of all the CIs, 0.5% (n = 8) before and 0.6% (n = 11) after guideline adaptation included more than 200 contacts and because these were outliers, they were excluded from further analysis. Of the 3,192 index patients, 3,088, 1,335, and 365 had close, casual and community contacts eligible for CI, respectively. The characteristics of the index patients were comparable in both periods: about 35% had smear positive PTB, about 90% were passively identified and about 80% were of non-Dutch origin (Supplementary Table S5).

### Number of contacts invited per contact investigation

Before the guideline adaptation, 27,187 (median 6 per CI; IQR: 3–18) contacts were identified for CI; after the guideline adaptation, 21,056 (median 6 per CI; IQR: 3–15) were identified for CI. The number of casual contacts invited per CI decreased statistically significant from a median of 9 (IQR: 4–25) to a median of 8 (IQR: 3–20) (RR = 0.88; 95% CI: 0.79–0.98; p = 0.025) (Table 1). There was no decrease in the number of close and community contacts invited (Table 1). In all close, casual and community contacts, the number of contacts invited per CI was (marginally) larger for smear-positive index patients, Dutch index patients and patients belonging to a marginalised group (Table 1). For close and casual contacts, the number of contacts invited per CI was also larger for index patients younger than 15 years old (Table 1). For close contacts, the number of contacts invited per CI was larger for passively identified index patients (Table 1).

**Table 1:** Number of close, casual and community contacts invited per tuberculosis index patient by time period, demographic and patient characteristics, the Netherlands, 2011–2016 (n = 48,243)

Characteristics IP	Close contacts					Casual contacts					Community contacts				
	IP n	Contacts n	Median per IP (IQR)	aOR (95%CI)	p value	IP n	Contacts n	Median per IP (IQR)	aOR (95 %CI)	p value	IP n	Contacts n	Median per IP (IQR)	aOR (95 %CI)	p value
Total	3,088	20,649	4 (2–7)	NA		1,335	23,845	NA	NA		365	3,749	4 (2–9)	NA	
Period															
2011–2013	1,641	11,029	4 (2–7)	ref	NA	723	13,979	9 (4–25)	ref	NA	218	2,179	3 (2–8)	ref	NA
2014–2016	1,447	9,620	4 (2–7)	1.01 (0.94–1.09)	0.788	612	9,866	8 (3–20)	0.88 (0.79–0.98)	0.025	147	1,570	4 (2–12)	1.1(0.87–1.38)	0.424
Age (years)															
0–14	105	895	5 (3–8)	ref	NA	34	690	12 (3–26)	ref	NA	7	32	3 (2–6)	ref	NA
15–29	859	6,139	4 (2–8)	0.66 (0.53–0.82)	< 0.001	368	6,122	9 (4–22)	0.65 (0.45–0.94)	0.023	101	662	3 (1–6)	1.33 (0.56–3.15)	0.519
30–44	864	4,870	3 (2–6)	0.54 (0.43–0.67)	< 0.001	330	6,289	9 (3–24)	0.65 (0.45–0.94)	0.024	111	1,585	5 (2–18)	2.38 (1–5.63)	0.049
45–59	616	4,158	4 (2–6)	0.61 (0.49–0.76)	< 0.001	289	4,791	7 (4–21)	0.55 (0.38–0.8)	0.002	81	810	4 (2–8)	1.67 (0.7–3.97)	0.248
60–74	402	2,681	3 (1–8)	0.59 (0.47–0.75)	< 0.001	198	3,161	8 (3–21)	0.6 (0.41–0.88)	0.008	45	358	2 (1–5)	1.32 (0.54–3.21)	0.541
≥ 75	242	1,906	4 (2–10)	0.73 (0.56–0.93)	0.012	116	2,792	12 (4–31.5)	0.96 (0.64–1.43)	0.849	20	302	8 (4–18)	3.24 (1.24–8.49)	0.017
Sex^a^															
Male	1,740	12,096	4 (2–8)	NA		835	15,198	9 (4–24)	NA		232	2,406	4 (2–10.5)	NA	
Female	1,348	8,553	4 (2–7)	NA		500	8,647	8 (3–20)	NA		133	1,343	4 (2–8)	NA	
Infectiousness IP															
SM + PTB	1,079	11,428	6 (3–12)	ref	NA	866	17,678	12 (5–26)	ref	NA	293	3,213	4 (2–10)	ref	NA
SM-/C + PTB	742	4,280	4 (2–6)	0.55 (0.5–0.61)	< 0.001	324	4,705	6 (3–16)	0.68 (0.6–0.78)	< 0.001	56	483	3 (1–7.5)	0.71 (0.51–0.99)	0.043
SM-/C - PTB	214	997	3 (2–5)	0.44 (0.37–0.51)	< 0.001	39	602	4 (2–13)	0.61 (0.43–0.85)	0.004	5	16	3 (2–3)	0.31 (0.1–0.91)	0.033
EPTB	1,053	3,944	3 (2–4)	0.36 (0.33–0.39)	< 0.001	106	860	4 (2–8)	0.41 (0.33–0.51)	< 0.001	11	37	2 (2–5)	0.39 (0.2–0.79)	0.008
Ethnicity															
Dutch	629	5,205	4 (2–9)	ref	NA	343	8,004	12 (5–31)	ref	NA	93	1,429	4 (2–20)	ref	NA
Non-Dutch	2,459	15,444	4 (2–7)	0.86 (0.78–0.95)	0.002	992	15,841	8 (3–20)	0.69 (0.61–0.79)	< 0.001	272	2,320	4 (2–8)	0.6 (0.46–0.77)	< 0.001
Case finding															
Active	280	1,443	4 (2–7)	ref	NA	111	1,999	8 (2–22)	NA		26	325	4 (1–22)	NA	
Passive	2,808	19,206	4 (2–7)	1.37(1.19- 1.58)	< 0.001	1,224	21,846	9 (4–23)	NA		339	3,424	4 (2–9)	NA	
Marginalised group															
No	2,958	19,390	4 (2–7)	ref	NA	1,222	21,254	8 (3–22)	ref	NA	323	2,979	4 (2–8)	ref	NA
Yes	130	1,259	5 (2–10)	1.19 (0.98–1.44)	0.08	113	2,591	13 (4–30)	1.25 (1.02–1.54)	0.031	42	770	5.5 (2–30)	1.66 (1.18–2.34)	0.004

aOR: adjusted odds ratio; C: culture; CI: confidence interval; EPTB: extra-pulmonary TB; IP: index patient; IQR: interquartile range; NA: not applicable; ref: reference; SM: smear microscopy; TB: tuberculosis.

^a ^Covariate with a univariate p value > 0.2 not included in the multivariable model.

### Appropriate scale-up

The proportion of CIs appropriately not scaled-up to casual contacts given the absence of evidence for transmission among close contacts increased from 70.1% (772/1,102) between 2011 and 2013 to 72.6% (640/882) between 2014 and 2016. This increase was not statistically significant (RR = 1.17; 95% CI: 0.93–1.47; p = 0.177) (Table 2). The proportion of CIs appropriately not scaled-up to community contacts increased statistically significantly, from 74.3% (361/486) between 2011 and 2013 to 84.6% (235/384) between 2014 and 2016 (RR = 1.81; 95% CI: 1.28–2.57; p = 0.001) (Table 2). Appropriate scaling up from close to casual contacts was independently associated with smear negative pulmonary or extrapulmonary TB disease and non-Dutch ethnicity, and appropriate scaling up from casual to community contacts was independently associated with smear negative pulmonary or extrapulmonary TB disease (Table 2).

**Table 2:** Appropriately scaled-up contact investigation from close to casual contacts and from casual to community contacts for tuberculosis index patients given documented transmission by period and patient characteristics, the Netherlands, 2011–2016 (n = 3,088)

Characteristics IP	Close to casual						Casual to community					
	IP with close contacts	No transmission among close contacts n	Appropriately no scale-up n	Appropriately no scale-up %	aOR (95% CI)	p value	IP with casual contacts	No transmission among casual contacts n	Appropriately no scale-up n	Appropriately no scale-up %	aOR (95% CI)	p value
Period												
2011–2013	1,641	1,102	772	70.1	ref	NA	723	486	361	74.3	NA	
2014–2016	1,447	882	640	72.6	1.17 (0.93–1.47)	0.177	612	384	325	84.6	1.81 (1.28–2.57)	< 0.001
Age (years)												
0–14	105	45	34	75.6	ref	NA	34	21	18	85.7	NA	
15–29	859	508	364	71.7	1.37 (0.61–3.06)	0.446	368	249	196	78.7		
30–44	864	568	442	77.8	1.54 (0.69–3.43)	0.296	330	201	154	76.6		
45–59	616	392	284	72.4	1.38 (0.61–3.13)	0.437	289	190	148	77.9		
60–74	402	287	178	62.0	0.86 (0.38–1.97)	0.728	198	134	106	79.1		
≥ 75	242	184	110	59.8	0.97 (0.41–2.27)	0.939	116	75	64	85.3		
Sex^a^												
Male	1,740	1,099	755	68.7	NA		835	532	415	78.0	NA	
Female	1,348	885	657	74.2	NA		500	338	271	80.2	NA	
Infectiousness IP												
SM + PTB	1,079	453	159	35.1	ref	NA	866	528	386	73.1	ref	NA
SM-/C + PTB	742	526	338	64.3	3.3 (2.53–4.31)	< 0.001	324	232	202	87.1	2.39 (1.55–3.67)	< 0.001
SM-/C- PTB	214	182	157	86.3	12.33 (7.69–19.78)	< 0.001	39	28	24	85.7	2.02 (0.69–5.98)	0.202
EPTB	1,053	823	758	92.1	20.73 (15.03–28.59)	< 0.001	106	82	74	90.2	3.32 (1.56–7.07)	0.002
Ethnicity												
Dutch	629	438	270	61.6	ref	NA	343	239	187	78.2	NA	
Non-Dutch	2,459	1,546	1,142	73.9	1.39(1.05–1.85)	0.021	992	631	499	79.1	NA	
Case finding^a^												
Active	280	199	144	72.4	NA		111	82	68	82.9	NA	
Passive	2,808	1,785	1,268	71.0	NA		1,224	788	618	78.4	NA	
Marginalised group^a^												
No	2,958	1,913	1,381	72.2	NA		1,222	804	636	79.1	NA	
Yes	130	71	31	43.7	NA		113	66	50	75.8	NA	

aOR: adjusted odds ratio; C: culture; CI: confidence interval; EPTB: extra-pulmonary TB; IP: index patient; IQR: interquartile range; NA: not applicable; ref: reference; SM: smear microscopy; TB: tuberculosis.

^a^ Covariate with a univariate p value > 0.2 not included in the multivariable model.

### Tuberculosis testing coverage

The overall TB testing coverage increased from 85.8% (23,334/27,187) between 2011 and 2013 to 88.9% (18,723/21,056) between 2014 and 2016. The testing coverage increased statistically significantly among casual contacts, from 83% to 88% (OR = 1.43; 95% CI: 1.18–1.74; p < 0.001) (Table 3). The testing coverage increased borderline statistically significantly for close contacts, from 90% to 92% (OR = 1.18; 95% CI: 0.98–1.42; p = 0.08) (Table 3). The testing coverage did not change for community contacts (Table 3). For close and casual contact, the testing coverage was higher among contacts of index patients younger than 15 years old. For casual contacts, the testing coverage was higher for contacts of index patients of Dutch origin and passively detected index patients but not for socially marginalised risk groups (Table 3). For community contacts, coverage of TB testing was higher among contacts of index patients detected passively (Table 3).

**Table 3:** Tuberculosis testing coverage among close, casual and community contacts, of tuberculosis index patients by period and patient characteristics, the Netherlands, 2011–2016 (n = 48,243)

Characteristics IP	Close contacts						Casual contacts						Community contacts					
	IP n	Contacts n	TB tested n	TB tested %	aOR (95%CI)	p value	IP n	Contacts n	TB tested n	TB tested %	aOR (95 % CI)	p value	IP n	Contacts n	TB tested n	TB tested %	aOR (95 % CI)	p value
Total	3,088	20,649	18,739	91	NA		1,335	23,845	20,238	85	NA		365	3,749	3,080	82	NA	
Period																		
2011–2013	1,641	11,029	9,938	90	ref	NA	723	13,979	11,604	83	ref	NA	218	2,179	1,792	82	ref	NA
2014–2016	1,447	9,620	8,801	92	1.18 (0.98–1.42)	0.08	612	9,866	8,634	88	1.31 (1.09–1.57)	0.004	147	1,570	1,288	82	1.19 (0.83–1.7)	0.34
Age (years)																		
0–14	105	895	860	96	ref	NA	34	690	634	92	ref	NA	7	32	28	88	ref	NA
15–29	859	6,139	5,477	89	0.34 (0.19–0.61)	< 0.001	368	6,122	5,091	83	0.41 (0.28–0.59)	< 0.001	101	662	529	80	0.61 (0.2–1.85)	0.388
30–44	864	4,870	4,427	91	0.41 (0.23–0.74)	0.003	330	6,289	5,275	84	0.47 (0.32–0.69)	< 0.001	111	1,585	1,344	85	0.71 (0.24–2.14)	0.545
45–59	616	4,158	3,761	91	0.39 (0.22–0.70)	0.002	289	4,791	4,163	87	0.54 (0.36–0.81)	0.003	81	810	615	76	0.37 (0.12–1.14)	0.083
60–74	402	2,681	2,446	91	0.44 (0.24–0.79)	0.006	198	3,161	2,760	87	0.51 (0.32–0.82)	0.005	45	358	305	85	0.69 (0.23–2.04)	0.502
≥ 75	242	1,906	1,768	93	0.53 (0.29–0.97)	0.039	116	2,792	2,315	83	0.36 (0.23–0.57)	< 0.001	20	302	259	86	0.79 (0.24–2.62)	0.694
Sex^a^																		
Male	1,740	12,096	10,933	90	NA		835	15,198	12,781	84	NA		232	2,406	1,941	81	NA	
Female	1,348	8,553	7,806	91	NA		500	8,647	7,457	86	NA		133	1,343	1,139	85	NA	
Infectiousness IP																		
SM + PTB	1,079	11,428	10,311	90	ref	NA	866	17,678	15,030	85	ref	NA	293	3,213	2,668	83	ref	NA
SM-/C + PTB	742	4,280	3,871	90	0.98 (0.78–1.22)	0.832	324	4,705	4,039	86	1.04 (0.82–1.31)	0.762	56	483	379	79	0.65 (0.37–1.13)	0.127
SM-/C- PTB	214	997	904	91	0.94 (0.64–1.38)	0.768	39	602	438	73	0.45 (0.21–0.94)	0.034	5	16	11	69	0.28 (0.12–0.68)	0.004
EPTB	1,053	3,944	3,653	93	1.32 (1.04–1.67)	0.023	106	860	731	85	0.86 (0.64–1.16)	0.318	11	37	22	60	0.29 (0.13–0.65)	0.002
Ethnicity^a^																		
Dutch	629	5,205	4,791	92	NA		343	8,004	6,952	87	ref	NA	93	1,429	1,217	85	ref	NA
Non-Dutch	2,459	15,444	13,948	90	NA		992	15,841	13,286	84	0.8 (0.65–0.98)	0.035	272	2,320	1,863	80	0.7 (0.48–1.03)	0.074
Case finding^a^																		
Active	280	1,443	1,279	89	NA		111	1,999	1,509	76	ref	NA	26	325	194	60	ref	NA
Passive	2,808	19,206	17,460	91	NA		1,224	21,846	18,729	86	1.66 (1.2–2.27)	0.002	339	3,424	2,886	84	2.85(1.51–5.39)	0.001
Marginalised group^a^																		
No	2,958	19,390	17,641	91	NA		1,222	21,254	18,229	86	ref	NA	323	2,979	2,500	84	ref	NA
Yes	130	1,259	1,098	87	NA		113	2,591	2,009	78	0.58 (0.43–0.8)	0.001	42	770	580	75	0.66 (0.43–1.01)	0.056

aOR: adjusted odds ratio; C: culture; CI: confidence interval; EPTB: extra-pulmonary TB; IP: index patient; IQR: interquartile range; NA: not applicable; ref: reference; SM: smear microscopy; TB: tuberculosis.

^a^ Covariate with a univariate p value > 0.2 not included in the multivariable model.

### Latent tuberculosis infection testing coverage

The overall LTBI testing coverage increased from 73% (19,964/27,187) between 2011 and 2013 to 85% (17,843/21,056) between 2014 and 2016. The LTBI testing coverage increased statistically significantly among close contacts (75.7% vs 86.0%; OR = 2.0; 95% CI: 1.7–2.4; p < 0.001), casual contacts (72.4% vs 84.2%) (OR = 1.9 95% CI: 1.7–2.3; p < 0.001) and community contacts (69.0% vs 80.6%) (OR = 2.2; 95% CI: 1.5–3.0; p < 0.001) (Table 4). In all three groups, the coverage of LTBI testing was statistically significantly higher among contacts of index patients younger than 15 years old (Table 4). For close and casual contacts, the coverage of LTBI testing was statistically significantly higher among contacts of Dutch index patients and index patients not belonging to a socially marginalised group (Table 4). For close and community contacts, the coverage of LTBI testing was statistically significantly higher among contacts of index patients with sputum positive pulmonary TB (Table 4).

**Table 4:** Latent tuberculosis infection testing coverage among close, casual, and community contacts of tuberculosis index patients by period and patient characteristics, the Netherlands, 2011–2016 (n = 48,243)

Characteristics IP	Close contacts						Casual contacts						Community contacts					
	IP n	Contacts n	LTBI tested n	LTBI tested %	aOR (95 % CI)	p value	IP n	Contacts n	LTBI tested n	LTBI tested %	aOR (95 % CI)	p value	IP n	Contacts n	LTBI tested n	LTBI tested %	aOR (95 % CI)	p value
Total	3,088	20,649	16,618	81	NA		1,335	23,845	18,419	77	NA		365	3,749	2,770	74	NA	
Period																		
2011–2013	1,641	11,029	8,344	76	ref	NA	723	13,979	10,116	72	ref	NA	218	2,179	1,504	69	ref	NA
2014–2016	1,447	9,620	8,274	86	2.00 (1.69–2.36)	< 0.001	612	9,866	8,303	84	1.94 (1.65–2.28)	< 0.001	147	1,570	1,266	81	2.15 (1.52–3.03)	< 0.001
Age (years)																		
0–14	105	895	801	90	ref	NA	34	690	601	87	ref	NA	7	32	28	88	ref	NA
15–29	859	6,139	4,805	78	0.39 (0.25–0.6)	< 0.001	368	6,122	4,631	76	0.48 (0.32–0.71)	< 0.001	101	662	488	74	0.49 (0.2–1.18)	0.11
30–44	864	4,870	3,895	80	0.45 (0.29–0.71)	0.001	330	6,289	4,753	76	0.52 (0.34–0.78)	0.002	111	1,585	1,232	78	0.51 (0.21–1.23)	0.135
45–59	616	4,158	3,376	81	0.47 (0.3–0.74)	0.001	289	4,791	3,796	79	0.55 (0.36–0.83)	0.005	81	810	556	69	0.29 (0.12–0.75)	0.01
60–74	402	2,681	2,183	81	0.45 (0.28–0.72)	0.001	198	3,161	2,454	78	0.44 (0.28–0.68)	< 0.001	45	358	247	69	0.36 (0.14–0.88)	0.026
≥ 75	242	1,906	1,558	82	0.41 (0.26–0.67)	< 0.001	116	2,792	2,184	78	0.44 (0.28–0.7)	< 0.001	20	302	219	73	0.51 (0.19–1.36)	0.176
Sex^a^																		
Male	1,740	12,096	9667	80	NA		835	15,198	11,566	76	NA		232	2,406	1,738	72	NA	
Female	1,348	8,553	6951	81	NA		500	8,647	6,853	79	NA		133	1,343	1,032	77	NA	
Infectiousness IP																		
SM + PTB	1,079	11,428	9,415	82	ref	NA	866	17,678	13,623	77	NA		293	3,213	2,416	75	ref	NA
SM-/C + PTB	742	4,280	3,414	80	0.79 (0.64–0.96)	0.017	324	4,705	3,734	79	NA		56	483	325	67	0.62 (0.38–1.02)	0.06
SM-/C-PTB	214	997	797	80	0.67 (0.5–0.9)	0.008	39	602	408	68	NA		5	16	11	69	0.5 (0.25–0.99)	0.048
EPTB	1,053	3,944	2,992	76	0.66 (0.55–0.79)	< 0.001	106	860	654	76	NA		11	37	18	49	0.33 (0.17–0.64)	0.001
Ethnicity																		
Dutch	629	5,205	4,491	86	ref	NA	343	8,004	6,506	81	ref	NA	93	1,429	1,075	75	NA	NA
Non-Dutch	2,459	15,444	12,127	79	0.59 (0.48–0.71)	< 0.001	992	15,841	11,913	75	0.7 (0.58–0.84)	< 0.001	272	2,320	1,695	73	NA	NA
Case finding^a^																		
Active	280	1,443	1,118	78	NA		111	1,999	1,256	63	ref	NA	26	325	181	56	ref	NA
Passive	2,808	19,206	15,500	81	NA		1,224	21,846	17,163	79	1.79 (1.36–2.36)	< 0.001	339	3,424	2,589	76	2.85 (1.73–4.7)	< 0.001
Marginalised group^a^																		
No	2,958	19,390	15,658	81	ref	NA	1,222	21,254	16,672	78	ref	NA	323	2,979	2,219	75	NA	
Yes	130	1,259	960	76	0.66 (0.46–0.95)	0.024	113	2,591	1,747	67	0.64 (0.64–0.84)	0.001	42	770	551	72	NA	

aOR: adjusted odds ratio; C: culture; EPTB: extra-pulmonary TB; IP: index patient; IQR: interquartile range; NA: not applicable; ref: reference; SM: smear microscopy; TB: tuberculosis.

^a ^Covariate with a univariate p value > 0.2 not included in the multivariable model.

### Tuberculosis yield

The yield of active TB among contacts increased from 0.70% (164/23,334) to 0.73% (136/18,723) after guideline adaptation. The TB yield increased statistically significantly among casual contacts, from 0.17% to 0.28% (OR = 2.0; 95% CI: 1.0–3.9; p = 0.045) (Table 5). There was no statistically significant difference in the TB yield among close contacts (1.4% vs 1.3%) (OR = 0.97; 95% CI: 0.68–1.4; p = 0.854) (Table 6). The yield among community contacts (0.11% vs 0%) could not be compared statistically as too few patients (n = 2) were identified among this group. In the stratified analysis per contact group, characteristics of the index patient independently associated with a higher yield of TB diagnosis among close contacts were age < 30 years, sputum positive pulmonary TB and non-Dutch ethnicity (Table 5). For casual contacts, male sex was the only characteristic of the index patient associated with a higher yield of contact investigation, and non-Dutch ethnicity was borderline statistically significant (Table 5).

**Table 5:** Tuberculosis yield among close, casual and community contacts of tuberculosis index patients by period and patient characteristics, the Netherlands, 2011–2016 (n = 42,057)

Characteristics IP	Close contacts						Casual contacts						Community contacts^a^			
	IP n	TB tested n	TB yield n	TB yield %	aOR (95 % CI)	p value	IP n	TB tested n	TB yield n	TB yield %	aOR (95 % CI)	p value	IP n	TB tested n	TB yield n	TB yield %
Total	3,088	18,739	254	1.4	NA		1,335	20,238	44	0.2	NA		365	3,080	2	0.1
Period																
2011–2013	1,641	9,938	142	1.4	ref	NA	723	11,604	20	0.2	ref	NA	218	1,792	2	0.1
2014–2016	1,447	8,801	112	1.3	0.97 (0.68–1.37)	0.854	612	8,634	24	0.3	1.99 (1.02–3.89)	0.045	147	1,288	0	0
Age (years)																
0–14	105	860	23	2.7	ref	NA	34	634	2	0.3	ref	NA	7	28	0	0
15–29	859	5,477	112	2.0	0.52 (0.27–1.02)	0.058	368	5,091	9	0.2	0.51 (0.1–2.52)	0.406	101	529	0	0
30–44	864	4,427	66	1.5	0.4 (0.2–0.79)	0.008	330	5,275	21	0.4	1.14 (0.24–5.35)	0.867	111	1,344	2	0.2
45–59	616	3,761	32	0.9	0.23 (0.11–0.51)	< 0.001	289	4,163	7	0.2	0.44 (0.08–2.24)	0.32	81	615	0	0
60–74	402	2,446	16	0.7	0.19 (0.08–0.43)	< 0.001	198	2,760	5	0.2	0.51 (0.09–2.78)	0.437	45	305	0	0
≥ 75	242	1,768	5	0.3	0.09 (0.03–0.27)	< 0.001	116	2,315	0	0	NA	NA	20	259	0	0
Sex^b^																
Male	1,740	10,933	154	1.4	NA		835	12,781	36	0.3	ref	NA	232	1,941	0	0
Female	1,348	7,806	100	1.3	NA		500	7,457	8	0.1	0.37 (0.17–0.82)	0.014	133	1,139	2	0.2
Infectiousness IP																
SM + PTB	1,079	10,311	203	2.0	ref	NA	866	15,030	35	0.2	NA		293	2,668	2	0.1
SM-/C + PTB	742	3,871	23	0.6	0.32 (0.18–0.55)	< 0.001	324	4,039	8	0.2	NA		56	379	0	0
SM-/C-PTB	214	904	0	0	NA		39	438	1	0.2	NA		5	11	0	0
EPTB	1,053	3,653	28	0.8	0.35 (0.2–0.59)	< 0.001	106	731	0	0	NA		11	22	0	0
Ethnicity																
Dutch	629	4,791	36	0.8	ref	NA	343	6,952	8	0.1	ref	NA	93	1,217	0	0
Non-Dutch	2,459	13,948	218	1.6	1.82 (1.15–2.88)	0.01	992	13,286	36	0.3	2.24 (0.99–5.07)	0.053	272	1,863	2	0.1
Case finding																
Active	280	1,279	10	0.8	ref	NA	111	1,509	1	0.1	NA		26	194	0	0
Passive	2,808	17,460	244	1.4	2.06 (0.98–4.31)	0.056	1,224	18,729	43	0.2	NA		339	2,886	2	0.1
Marginalised group^b^																
No	2,958	17,641	238	1.3	NA		1,222	18,229	34	0.2	ref	NA	323	2,500	1	0.0
Yes	130	1,098	16	1.5	NA		113	2,009	10	0.5	2.17 (0.92–5.09)	0.076	42	580	1	0.2

aOR: adjusted odds ratio; C: culture; CI: confidence interval; EPTB: extra-pulmonary TB; IP: index patient; IQR: interquartile range; NA: not applicable; ref: reference; SM: smear microscopy; TB: tuberculosis.

^a ^Regression analyses not conducted because insufficient statistical power.

^b ^Covariate with a univariate p value > 0.2 not included in the multivariable model.

**Table 6:** Latent tuberculosis infection yield among close, casual and community contacts of tuberculosis index patients by period and patient characteristics, the Netherlands, 2011–2016 (n = 37,807)

Characteristics IP	Close contacts						Casual contacts						Community contacts					
	IP n	LTBI tested n	LTBI yield n	LTBI yield %	aOR (95% CI)	p value	IP n	LTBI tested n	LTBI yield n	LTBI yield %	aOR (95% CI)	p value	IP n	LTBI tested n	LTBI yield n	LTBI yield %	aOR (95% CI)	p value
Total	3,088	16,618	2327	14	NA		1,335	18,419	1,052	6	NA		365	2,770	121	4	NA	
Period																		
2011–2013	1,641	8,344	1,168	14	ref	NA	723	10,116	535	5	ref	NA	218	1,504	46	3	ref	NA
2014–2016	1,447	8,274	1,159	14	1.06 (0.91–1.24)	0.461	612	8,303	517	6	1.24 (1–1.54)	0.048	147	1,266	75	6	2 (1.25–3.18)	0.004
Age (years)																		
0–14	105	801	143	18	ref	NA	34	601	38	6	ref	NA	7	28	1	4	NA	
15–29	859	4,805	823	17	0.77 (0.51–1.16)	0.208	368	4,631	291	6	0.97 (0.49–1.91)	0.932	101	488	21	4		
30–44	864	3,895	611	16	0.73 (0.48–1.11)	0.139	330	4,753	317	7	1.15 (0.58–2.28)	0.68	111	1,232	61	5		
45–59	616	3,376	459	14	0.65 (0.42–1.01)	0.053	289	3,796	223	6	0.95 (0.48–1.88)	0.888	81	556	27	5		
60–74	402	2,183	199	9	0.43 (0.27–0.68)	< 0.001	198	2,454	118	5	0.75 (0.38–1.5)	0.419	45	247	4	2		
≥ 75	242	1,558	92	6	0.3 (0.18–0.49)	< 0.001	116	2,184	65	3	0.55 (0.26–1.14)	0.106	20	219	7	3		
Sex^a^																		
Male	1,740	9,667	1395	14	NA		835	11,566	758	7	ref	NA	232	1,738	80	5	NA	
Female	1,348	6,951	932	13	NA		500	6,853	294	4	0.6 (0.47–0.75)	< 0.001	133	1,032	41	4		
Infectiousness IP																		
SM + PTB	1,079	9,415	1602	17	ref	NA	866	13,623	846	6	ref	NA	293	2,416	108	4	NA	
SM-/C + PTB	742	3,414	341	10	0.55 (0.46–0.67)	< 0.001	324	3,734	154	4	0.7 (0.54–0.92)	0.011	56	325	12	4		
SM-/C- PTB	214	797	52	7	0.34 (0.22–0.53)	< 0.001	39	408	13	3	0.79 (0.39–1.59)	0.509	5	11	0	0		
EPTB	1,053	2,992	332	11	0.56 (0.47–0.68)	< 0.001	106	654	39	6	0.91 (0.53–1.55)	0.723	11	18	1	6		
Ethnicity																		
Dutch	629	4,491	415	9	ref	NA	343	6,506	247	4	ref	NA	93	1,075	35	3	NA	
Non-Dutch	2,459	12,127	1912	16	1.67 (1.34–2.07)	< 0.001	992	11,913	805	7	1.84 (1.43–2.36)	< 0.001	272	1,695	86	5		
Case finding^a^																		
Active	280	1,118	144	13	NA		111	1,256	50	4	ref	NA	26	181	10	6	NA	
Passive	2,808	15,500	2183	14	NA		1,224	17,163	1,002	6	2.01 (1.35–3)	0.001	339	2,589	111	4		
Marginalised group^a^																		
No	2,958	15,658	2151	14	NA		1,222	16,672	913	6	NA		323	2,219	97	4	NA	
Yes	130	960	176	18	NA		113	1,747	139	8	NA		42	551	24	4		

aOR: adjusted odds radio; C: culture; CI: confidence interval; EPTB: extra-pulmonary TB; IP: index patient; IQR: interquartile range; NA: not applicable; ref: reference; SM: smear microscopy; TB: tuberculosis.

^a^ Covariate with a univariate p value > 0.2 not included in the multivariable model.

### Latent tuberculosis infection yield

The yield of LTBI among contacts increased from 8.8% (1,749/19,964) between 2011 and 2013 to 9.8% (1,751/17,843) between 2014 and 2016. The yield of LTBI increased statistically significantly for casual (5.3% vs 6.2%) (OR = 1.2; 95% CI: 1.0–1.5; p = 0.048) (Table 6) as well as community contacts (3.1 vs 5.9%) (OR = 2.0; 95% CI: 1.6–3.2; p = 0.004) (Table 6). There was no statistically significant difference in the LTBI yield among close contacts (Table 6). In the stratified analysis per contact group, characteristics of the index patient independently associated with a higher yield for LTBI diagnosis among close contacts were age < 60 years, sputum positive pulmonary disease and non-Dutch ethnicity (Table 6). For casual contacts, independently associated characteristics were female sex, non-Dutch ethnicity and passive case finding. Smear-negative, culture-positive pulmonary TB was negatively associated with a higher LTBI yield (Table 6).

## Discussion

In this study, we showed that adapting CI guidelines with a stronger focus on the stone-in-the-pond principle and clear dissemination and training efforts may have resulted in more efficient CI and increased the overall relative TB and LTBI yield among contacts. The TB yield among close contacts (1.4%) did not change significantly and is comparable to other low burden, high-income countries [10-12]. The yield of TB among casual contacts of 0.4% (0.2–0.6%) increased statistically significantly and became comparable to the TB yield among causal contacts in other high-income countries [10].

CIs were more often appropriately scaled up from casual to community contacts, indicating an improved risk assessment of the TB contacts and stricter adherence to the stone-in-the-pond principle as recommended in the updated guidelines. As fewer contacts were screened, the relative TB yield increased.

To the authors’ knowledge, no studies have evaluated the yield of CI among community contacts. WHO guidelines do not recommend extending CI to community contacts [4]. However, for low burden countries, it is recommended to screen for LTBI and treat risk groups that have a high likelihood of recent TB transmission [3]. US guidelines state that ‘low-priority contacts’ may be included if resources permit and the programme meets its performance goals [13]. The United Kingdom (UK) guidelines apply the stone-in-the-pond principle but do not differentiate between casual and community contacts [14]. Between 2011 and 2016, two community contacts were identified with TB (60 per 100,000 community contacts investigated). Despite a low numeric yield, the identification of community contacts eligible for CI is compliant with the national criteria for a target group of active case findings for TB, which is defined as a population with a prevalence or annual incidence of 50 TB patients per 100,000 persons.

The relative yield of LTBI among casual and community contacts screened for LTBI was higher after guideline adaption. This increase possibly resulted from better LTBI testing coverage, which improves decision making about whether to scale up to the next priority group. This improved prioritisation may explain the increase in the median number of community contacts invited (from 3 to 4 contacts) although this was not statistically significant. The LTBI yield among close and casual contacts, however, remained lower compared with other high-income countries [15,16]. This difference may result from variations in background prevalence and CI policies regarding contact eligibility, enrolment and diagnostic tests.

Significantly more contacts of foreign-born TB patients were offered and accepted LTBI testing. This may contribute substantially to eliminating TB in the Netherlands. The number of foreign-born persons with LTBI notified to the NTR and identified through CI increased by 21% in the period 2014 to 2016 compared with 2011 to 2013 [17-21], and the number of Dutch-born TB contacts with LTBI decreased by 19%. According to the national surveillance report from 2018, 78% of the contacts identified with LTBI were provided tuberculosis preventive treatment (TPT); in 2017, 88% completed the treatment [1]. These percentages are in line with the European consensus on CI target proportions for infected contacts on TPT initiation (85%) and TPT completion (75%) [22].

Our study has a few limitations. The classification of the contact group is determined by the public health nurse based on an assessment of the intensity and frequency of the contact with the index patient. As the NTR data cannot be used to verify classification, there may have been some over- or underestimation of the true number invited, coverage and yield per contact group. However, given the reduction of the median number of casual contacts before and after the trainings, it is likely that the recommendations for classification were followed more accurately.

The NTR does not provide any characteristics of the individual contacts as contact data are aggregated per index patient. Hence changes in contact populations before and after the guideline adaption could not be analysed, which may have biased the TB and LTBI yield. Overall, the surveillance data registered in the NTR may not reflect all improvements achieved through the guideline adaption and the corresponding training activities. However, surveillance data show significant positive trends in CI outcomes and provide a basis for further investigations into CI practices.

### Conclusion

This study shows how the adherence to CI guidelines based on the stone-in-the-pond principle can be monitored and evaluated. Careful implementation of new recommendations through nationwide training, administrative support and regular evaluation strengthens the efficiency of conducting CIs without jeopardising the yield. This is likely to improve the cost-effectiveness of CI.

## Supplementary Data

421000 Supplementary Material
