# Supplementary material for: Tuberculosis contact investigation following the stone-in-the-pond principle in the Netherlands – Did adjusted guidelines improve efficiency?
Source: Euro Surveill. 2021 Nov 11;26(45):2001828. doi: 10.2807/1560-7917.ES.2021.26.45.2001828 (PMC8646980; doi:10.2807/1560-7917.ES.2021.26.45.2001828)
Supplement: Supplementary Material [file 20-01828_MULDER_Supplementary_material.pdf]

Supplementary Material

This supplementary material is hosted by *Eurosurveillance* as supporting information alongside the article 'Tuberculosis contact investigation following the stone-in-the-pond principle in the Netherlands – Did adjusted guidelines improve efficiency?', on behalf of the authors, who remain responsible for the accuracy and appropriateness of the content. The same standards for ethics, copyright, attributions and permissions as for the article apply. Supplements are not edited by *Eurosurveillance* and the journal is not responsible for the maintenance of any links or email addresses provided therein.

Supplement S1. Definition of contact priority circles according to stone-in-the-pond principle

- **First circle (ring) / close contacts:** are individuals with intimate and prolonged contact with an infectious index patient, e.g. household contacts or contacts who share the same working/living space for more than 48 cumulative hours; *This includes those who share the same breathing space with the index patient on a daily basis, such as members of the same household and persons who have spent more than 48 hours in the same confined room. This group also includes contacts with regular, prolonged contact with the source patient, who share breathing space but do not live in the same household or who have spent time with the source patient in a confined space, such as a car, sweat shop or prison cell. It may include those persons with short exposure times to direct face-to-face streams of air with a particularly high density of infectious droplet nuclei, such as may occur during bronchoscopy or otorhinolaryngeal examination of patients with hence untreated sputum smear-positive tuberculosis, and similar situations.*
- **Second circle (ring) / casual contacts:** are individuals with intimate and less prolonged (6-48 hours) or less intimate but prolonged (>48 hours) contact with an infectious index patient. *This includes those who have shared the same breathing space in a confined room repeatedly for short periods (cumulatively 6-48 hours) or those who have had prolonged contact in a larger confined space. These may include frequent visitors to the home, friends, relatives, school or classmates, colleagues at work or leisure contacts and members of a club or team or passengers in adjoining seats during aircraft travel of more than 8 hours.*
- **Third circle (ring) / community contacts:** are individuals with less intimate and less frequent, often undocumented contact with an infectious index patient. *This includes those who have spent less than 6-48 hours with the index patients and did not share direct breathing space: i.e. those living in the same community or attending the same school, sports club or workplace who may have had sporadic contact.*

Diagram to determine the risk of exposure of a contact taking into account whether contacts are considered close (1st ring), casual (2nd ring), or community (3rd ring) contacts.

		Duration		
**Size of the space**		Prolonged	Short	
		Daily or >48 hours	Weekly or 6-48 hours	Occasionally or 1-6 hours
Car	<5 m^3	Household and other close contacts 1st ring	Close or casual contacts* 1st ring or 2nd ring	Casual contacts** 2nd ring
Room	10-30 m^3	Household and other close contacts 1st ring	Casual contacts** 2nd ring	Casual contacts** 2nd ring
Class/office	100-200 m^3	Casual contacts** 2nd ring	Casual contacts ** 2nd ring	Community contacts *** 3rd ring
Large closed space	>200 m^3	Community contacts*** 3rd ring	Community contacts 3rd ring	Community contacts 3rd ring

- *Consider as 'close' when index patient has sputum smear positive TB
- ** Consider CI when there is evidence of transmission among 'close' contacts
- *** Consider CI when there is evidence of transmission among casual contacts in congregate settings such as schools

Supplementary Table S2. Estimated prevalence of latent tuberculosis infection in the Netherlands

<table>
<tr><th colspan="2">Dutch born</th><th colspan="4">Foreign born</th></tr>
<tr><th rowspan="2">Year of birth</th><th rowspan="2">LTBI prevalence *</th><th rowspan="2">Age group</th><th rowspan="2">Incidence in country of origin per 100.000**</th><th colspan="2">LTBI prevalence #</th></tr>
<tr><th>TST≥10mm</th><th>IGRA-positive</th></tr>
<tr><td>2010</td><td><0,1%</td><td rowspan="3"><18</td><td rowspan="2"><100</td><td rowspan="2">10%</td><td rowspan="2">4%</td></tr>
<tr><td>2000</td><td>0,2%</td></tr>
<tr><td>1990</td><td>0,3%</td><td>≥100</td><td>14%</td><td>8%</td></tr>
<tr><td>1980</td><td>0,5%</td><td rowspan="3">18-24</td><td rowspan="2"><100</td><td rowspan="2">24%</td><td rowspan="2">9%</td></tr>
<tr><td>1970</td><td>0,9%</td></tr>
<tr><td>1960</td><td>1,8%</td><td>≥100</td><td>32%</td><td>19%</td></tr>
<tr><td>1950</td><td>5%</td><td rowspan="3">25-60</td><td rowspan="2"><100</td><td rowspan="2">47%</td><td rowspan="2">19%</td></tr>
<tr><td>1940</td><td>16%</td></tr>
<tr><td>1930</td><td>41%</td><td>≥100</td><td>49%</td><td>27%</td></tr>
<tr><td>1920</td><td>70%</td><td>≥60</td><td colspan="3">unknown</td></tr>
</table>

* The estimated infection prevalence is based on an extrapolation of the infection prevalence among Dutch military recruits with a tuberculin skin test (TST) ≥10mm (2). In the period after 1990 the annual infection risk was estimated as 0,01%.

** Non-Western countries: WHO estimated incidence.

[#] LTBI prevalence based on positive interferon gamma assay (IGRA)

Supplementary Table S3. Screening algorithm for identified contacts of tuberculosis patients in the Netherlands

<table>
<tr><th rowspan="2">Priority</th><th rowspan="2">Contact group</th><th colspan="2">Timing and screening tests</th></tr>
<tr><th>< 1-2 weeks after diagnosis index patient</th><th>< 8-12 weeks after diagnosis index patient</th></tr>
<tr><td rowspan="3">High (close contacts)</td><td>Symptomatic</td><td rowspan="2">Chest X-ray and TST or IGRA</td><td rowspan="3">Chest X-ray and TST or IGRA</td></tr>
<tr><td>Vulnerable</td></tr>
<tr><td>Other 1st ring</td><td rowspan="2">Optional* Chest X-ray and TST or IGRA</td></tr>
<tr><td rowspan="2">Medium (casual contacts)</td><td>Vulnerable</td><td>Optional** Chest X-ray and TST or IGRA</td></tr>
<tr><td>Other 2nd ring</td><td>Not eligible at this time</td><td>In case of transmission: TST or IGRA, chest X-ray in those with positive test</td></tr>
<tr><td>Low (community contacts)</td><td></td><td>Not eligible at this time</td><td>Optional in case of transmission in 2d ring: TST or IGRA, chest X-ray in those with positive test</td></tr>
</table>

* in smear positive PTB patients with expected period of infectiousness > 8 weeks

** always when transmission detected among high priority contacts

Supplementary Figure S4. Flow diagram of 3,192 index patients with contact investigation results in the period 2011-2016 in the Netherlands.

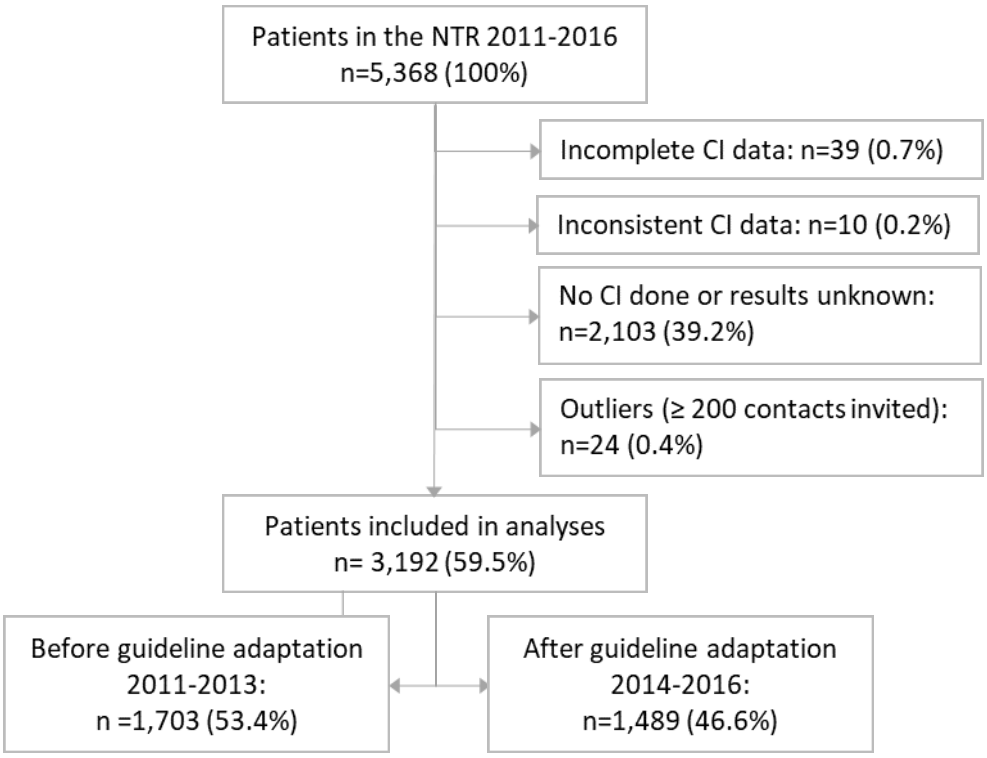

CI: contact investigation; NTR: National Tuberculosis Register

Supplementary Table S5. Characteristics of index patients registered in the National Tuberculosis Register in the periods 2011-2013 and 2014-2016 in the Netherlands

	**Total**		**2011-2013**		**2014-2016**	
**Index patient characteristics**	n	%	n	%	n	%
**Total**	3,192	100%	1,703	100%	1,489	100%
**Age**						
0-14	105	3%	52	3%	53	4%
15-29	881	28%	478	28%	403	27%
30-44	891	28%	483	28%	408	27%
45-59	648	20%	348	20%	300	20%
60-74	416	13%	207	12%	209	14%
75+	251	8%	135	8%	116	8%
**Sex**						
Male	1,817	57%	949	56%	868	58%
Female	1,375	43%	754	44%	621	42%
**Infectiousness**						
Smear-positive pulmonary TB	1,131	35%	621	36%	510	34%
Smear-negative, culture-positive pulmonary TB	772	24%	400	23%	372	25%
Smear-negative, culture-negative pulmonary TB	217	7%	94	6%	123	8%
Extrapulmonary TB	1,072	34%	588	35%	484	33%
**Ethnicity**						
Dutch	652	20%	335	20%	317	21%
Non-Dutch	2,540	80%	1368	80%	1172	79%
**Case finding**						
Active	306	10%	150	9%	156	10%
Passive	2,886	90%	1553	91%	1,333	90%
**Marginalized group**						
No	3,044	95%	1624	95%	1,420	95%
Yes	148	5%	79	5%	69	5%

TB: Tuberculosis
